# Measuring health disparities: a comparison of absolute and relative disparities

**DOI:** 10.7717/peerj.1438

**Published:** 2015-11-24

**Authors:** Ramal Moonesinghe, Gloria L.A. Beckles

**Affiliations:** 1 Office of Minority Health and Health Equity, Centers for Disease Control and Prevention, Atlanta, GA, United States; 2 National Center for Chronic Disease Prevention and Health Promotion, Centers for Disease Control and Prevention, Atlanta, GA, United States

**Keywords:** Health disparities, Absolute disparity, Relative disparity

## Abstract

Monitoring national trends in disparities in different diseases could provide measures to evaluate the impact of intervention programs designed to reduce health disparities. In the US, most of the reports that track health disparities provided either relative or absolute disparities or both. However, these two measures of disparities are not only different in scale and magnitude but also the temporal changes in the magnitudes of these measures can occur in opposite directions. The trends for absolute disparity and relative disparity could move in opposite directions when the prevalence of disease in the two populations being compared either increase or decline simultaneously. If the absolute disparity increases but relative disparity declines for consecutive time periods, the absolute disparity increases but relative disparity declines for the combined time periods even with a larger increase in absolute disparity during the combined time periods. Based on random increases or decreases in prevalence of disease for two population groups, there is a higher chance the trends of these two measures could move in opposite directions when the prevalence of disease for the more advantaged group is very small relative to the prevalence of disease for the more disadvantaged group. When prevalence of disease increase or decrease simultaneously for two populations, the increase or decrease in absolute disparity has to be sufficiently large enough to warrant a corresponding increase or decrease in relative disparity. When absolute disparity declines but relative disparity increases, there is some progress in reducing disparities, but the reduction in absolute disparity is not large enough to also reduce relative disparity. When evaluating interventions to reduce health disparities using these two measures, it is important to consider both absolute and relative disparities and consider all the scenarios discussed in this paper to assess the progress towards reducing or eliminating health disparities.

## Introduction

Disparities in health status and health care in the United States have been widely examined. However, many of these disparities have increased within the last 50 years (Institute of Medicine, 2002). There are several provisions in the Section 4302 of the Patient Protection and Affordable Care Act (ACA) to improve data collection and monitor trends in health disparities in federally funded programs. These provisions in the ACA could raise awareness among policymakers and the public about the existence and persistence of health disparities and the need for interventions to reduce disparities (Andrulis et al., 2010). Disparities in health and health care are addressed in the overarching goals and objectives for health promotion and disease prevention included in the nation’s Healthy People Initiative. In *Healthy People 2000*, “the overarching goal was to reduce health disparities among Americans” and in *Healthy People 2010*, “the overarching goal was to eliminate, not just reduce, health disparities” (US Department of Health and Human Services, 2015). The *Healthy People 2020* initiative will assess and track health disparities in “rates of death, chronic and acute diseases, injuries, and other health-related behaviors for subpopulations defined by race-ethnicity, gender identity, sexual orientation, disability status or special health care needs, and geographic location.” The Department of Health and Human Services (HHS) National Disparities Action Plan operationalizes the national goals in the *Healthy People 2020* initiative and increases its potential to be effective by using key provisions of the ACA and other cutting edge initiatives (US Department of Health and Human Services, 2011). Only few studies have attempted to estimate the economic burden of health disparities, and evidence of effective and cost-effective interventions that reduce the magnitude of disparities is scarce (LaVeist, Gaskin & Richard, 2011; Lorenc et al., 2013).

Monitoring national and subnational trends in disparities in different diseases could provide measures to evaluate the impact of intervention programs designed to reduce health disparities. Keppel, Pearcy & Heron (2010) examined changes in relative disparities between racial/ ethnic populations for the five leading causes of death in the United States from 1990 to 2006. Harper et al. (2008a) presented seven different disparity measures and applied them to US lung cancer rates from 1992 to 2006. Others found that their different summary measures provided different answers to the question whether disparity had increased or decreased (Messer, 2008; Bhopal, 2008). In the US, most of the reports that track health disparities reported on either relative or absolute disparities or both (Centers for Disease Control and Prevention, 2013; Agency for Health Care Research and Quality, 2013). However, when trends in health outcomes in different population groups are presented in line graphs or bar charts, these graphics only *illustrate* the pattern of secular change in absolute disparities.

**Figure 1:**
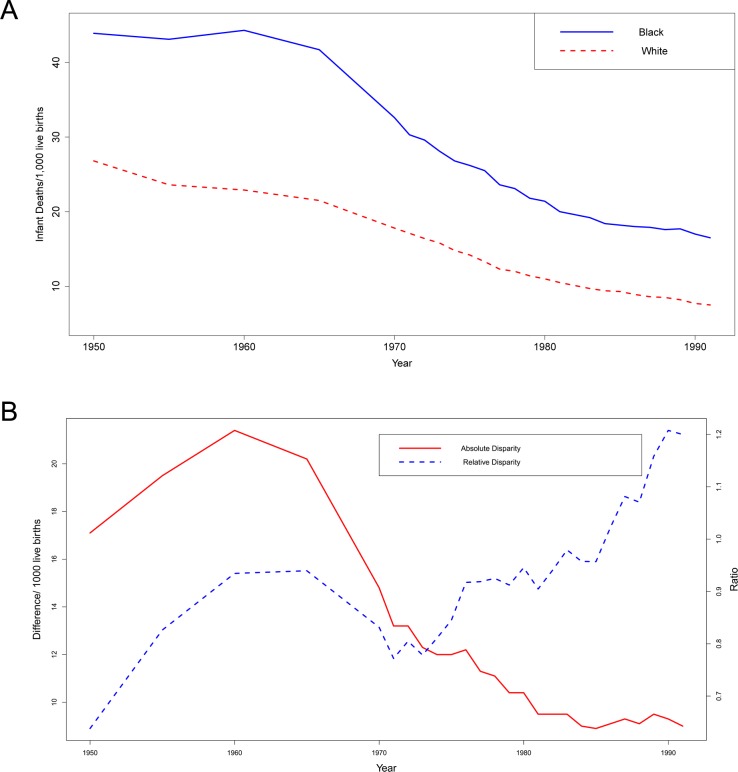
(A) Infant deaths per 1,000 live births from 1950 to 1991 in Black and White populations. (B) The black–white absolute disparity and relative disparity in infant deaths from 1950 to 1991.

Because absolute and relative disparities are measured on different scales, they are not only different in magnitude but also the temporal changes in the magnitudes of these measures can occur in opposite directions. For example, the magnitude of absolute and relative Black–White disparities in infant mortality rates in the US changed in opposite directions during the twentieth century (Lynch & Harper, 2005). Figure 1A shows that the infant deaths per 1,000 live births declined from 1950 to 1991 in both the Black and White populations. The size of the black–white absolute disparity decreased by almost half (46.8%) from 17.1 deaths/1,000 live births in 1950 to 9.1 deaths/ 1,000 live births in 1988 whereas, the size of the relative black–white disparity increased 68.3% from 0.64 to 1.07 during the same period (Fig. 1B). It has been shown that absolute disparities tend to be low when overall rates are high or low and tend to be high when rates are intermediate. On the other hand, relative disparities tend to be higher when overall rates are low (Houweling et al., 2007). In this paper, we consider all the possible scenarios for prevalence of disease for two population groups and analyze the trends in absolute and relative disparities for the two groups. We also provide conditions for which the changes in absolute and relative disparities move in opposite directions.

## Materials and Methods

Let *p* and *q* be the prevalence of a disease in populations Group 1 and Group 2, respectively at time *t*_0_. We assume that *p* > *q* or that Group 2 is the more socially advantaged compared to Group 1 and consider the more advantaged group as the reference group for estimating disparities between the two populations. Let *p* + *δp* and *q* + *δq* be the prevalence of disease for the two population groups at time *t*_1_ (*t*_1_ > *t*_0_). We also assume that 0 < |*δp*| < *p* and 0 < |*δq*| < *q* or the change in the prevalence of the disease from time *t*_0_ to time *t*_1_ is less than the prevalence of disease at time *t*_0_ for both population groups. The difference between the absolute disparity at time *t*_0_ and at time *t*_1_ is given by *δp* − *δq*. The difference between the relative disparity at time *t*_0_ and at time *t*_1_ is given by (*p* + *δp*)/(*q* + *δq*) − *p*/*q* = (*qδp* − *pδq*)/*q*(*q* + *δq*). The relative disparity between the two populations increases or decreases from time *t*_0_ to *t*_1_ depending on whether *qδp* > *pδq* or *qδp* < *pδq* or equivalently whether (*δp* − *δq*) > (*p* − *q*) *δq*/*q* or (*δp* − *δq*) < (*p* − *q*) *δq*/*q*.

First we consider increases in the prevalence of disease in each population group (*δp* > 0 and *δq* > 0). Suppose the absolute disparity in prevalence declines between time *t*_0_ and time *t*_1_, the change in the absolute disparity (*δp* − *δq*) will be <0. Then (*δp* − *δq*) < 0 < (*p* − *q*) *δq*/*q*, or the relative disparity between the two populations declines. In other words, a decline in absolute disparity leads to a decline in relative disparity. If the absolute disparity increases, i.e., (*δp* − *δq*) > 0, there are two possible scenarios: (1) 0 < (*δp* − *δq*) < (*p* − *q*) *δq*/*q*, in which case the relative disparity declines, and (2) 0 < (*p* − *q*) *δq*/*q* < (*δp* − *δq*), in which case the relative disparity increases. The converse of these statements also holds, i.e., if relative disparity increases then absolute disparity also increases, but a decline in relative disparity does not lead to a decline in absolute disparity if 0 < (*δp* − *δq*) < (*p* − *q*) *δq*/*q*.

Next, we consider the situation when *δp* < 0 and *δq* < 0 or the prevalence of disease in each population group declines from time *t*_0_ to *t*_1_. Since (*p* − *q*) *δq*/*q* < 0, an increase in absolute disparity, (*δp* − *δq*) > 0, leads to an increase in relative disparity, but a decline in absolute disparity leads to either a decline in relative disparity or an increase in relative disparity based on whether (*δp* − *δq*) < (*p* − *q*) *δq*/*q* < 0 or (*p* − *q*) *δq*/*q* < (*δp* − *δq*) < 0. The trend for absolute and relative disparities for this situation is identical to the previous situation (*δp* > 0 and *δq* > 0) when the trends are reversed.

If *δp* < 0 and *δq* > 0, i.e., the prevalence of disease for the more disadvantaged group declines but the prevalence of disease for the more advantaged group increases, then (*δp* − *δq*) < 0 and (*p* − *q*) *δq*/*q* > 0 indicating that both absolute and relative disparities decline. On the other hand, if *δp* > 0 and *δq* < 0, i.e., the prevalence of disease for the more disadvantaged group increases but the prevalence of disease for the more disadvantaged group increases, then (*δp* − *δq*) > 0 and (*p* − *q*) *δq*/*q* < 0 indicating that both absolute and relative disparities increase.

The trends for absolute disparity and relative disparity could move in opposite directions when the prevalence of disease in each population group either increase or decline simultaneously. When the prevalence of disease increases or declines for each population group, the relative change of increase in prevalence from time *t*_0_ to *t*_1_, i.e., (*δp* − *δq*)/*δq*, has to be greater than the relative disparity at time *t*_0_, i.e., (*p* − *q*)/*q*, for both absolute and relative disparities to increase or decline.

Suppose the prevalence of disease in each population group increases from time *t*_0_ to *t*_2_ (*t*_2_ > *t*_1_ > *t*_0_) and the increases in prevalence from time *t*_1_ to *t*_2_ for population Group 1 and population Group 2 are given by *δp*_2_ and *δq*_2_ respectively (*δp* > 0, *δq* > 0, *δp*_2_ > 0, *δq*_2_ > 0). If the absolute disparity increases but the relative disparity declines from time *t*_0_ to *t*_1_ and from time *t*_1_ to *t*_2_ then 0 < (*δp* − *δq*) < (*p* − *q*) *δq*/*q* and 0 < (*δp*_2_ − *δq*_2_) < [(*p* + *δp*) − (*q* + *δq*)]*δq*_2_/(*q* + *δq*). The sum of increases in absolute disparity from time *t*_0_ to *t*_1_ and *t*_1_ to *t*_2_ is additive and is given by (*δp* − *δq*) + (*δp*_2_ − *δq*_2_). It can be shown that 0 < (*δp* − *δq*) + (*δp*_2_ − *δq*_2_) < (*p* − *q*)(*δq* + *δq*_2_)/*q* or the relative disparity declines from time *t*_0_ to *t*_2_ even when the absolute disparity increases in the same time period. This means that if the absolute disparity increases but relative disparity declines for consecutive time periods, the absolute disparity increases but relative disparity declines for the whole time period even with a larger increase in absolute disparity during the whole time period.

We derived the probability of the relative disparity of increase in prevalence from time *t*_0_ to *t*_1_ is larger than the relative disparity at time *t*_0_ as a function of *p* and *q* (0.5 ≥ *p* > *q*) when both *p* and *q* increase randomly. The increases in *p* and *q*, *δp* and *δq*, were assumed to have uniform distributions in the intervals (0, *p*) and (0, *q*) respectively. If *y* = *δp*/*δq* with *δp* ∼ *U*(0, *p*) and *δq* ∼ *U*(0, *q*), the probability density function of *y* is given by:

\begin{eqnarray*} f(y)=q/2 p\hspace{1em}\text{when }y\lt p/q,\qquad f(y)=p/2 q{y}^{2}\hspace{1em}\text{when }y> p/q,\text{and }f(y)=0\text{ otherwise}. \end{eqnarray*}

It can be shown that the conditional probability of (*δp* − *δq*) > (*p* − *q*) *δq*/*q* given that (*δp* − *δq*) > 0 is *p*/(2*p* − *q*). When *q* is close to *p*, this probability is almost 1.0 whereas when *q* is close to 0, this probability approaches 0.5. These results are identical when the prevalence of disease in both populations decline (*δp* < 0 and *δq* < 0).

## Results and Discussion

Table 1 gives the percent increase in absolute disparity, and the percent increase in relative disparity given that the absolute disparity has increased for random increases of prevalence of disease in each population group with different prevalence of disease. As expected, the probability of an increase in absolute disparity tends to 50% when the prevalence of disease in each group is almost identical, but this probability increases with increasing difference between the prevalence of the two groups. For example, when *p* = 0.2 and *q* = 0.19, the probability of an increase in absolute disparity is 53% whereas the probability of an increase in absolute disparity is almost 100% when *p* = 0.2 and *q* = 0.001. Given that the absolute disparity increases, the probability that the relative disparity also increases ((*δp* − *δq*) > (*p* − *q*) *δq*/*q*) declines with declining value of *q* relative to *p*. When *p* = 0.2 and *q* = 0.19, the probability of an increase in relative disparity given that absolute disparity increases is 95%, but when *p* = 0.2 and *q* = 0.001, this probability is only 50%. Since relative disparity also declines when absolute disparity declines, both relative and absolute disparity either increase or decline (i.e., do not move in opposite directions) 97% of the time when *p* = 0.2 and *q* = 0.19, but both relative and absolute disparity either increase or decline only 50% of the time when *p* = 0.2 and *q* = 0.001. This result shows when using these two disparity measures to analyze trends in disparity, there is a higher chance the trends could move in opposite directions when *q* is very small relative to *p*. This result holds even for small values of *p* and *q*. For example, when *p* = 0.002 and *q* = 0.0019, the chance of both relative and absolute disparity either increase or decline is identical to the situation when *p* = 0.2 and *q* = 0.19.

**Table 1:** Percent increase in absolute disparity, and the percent increase in relative disparity given that the absolute disparity has increased for random increases of prevalence of disease for two population groups with different prevalence of disease.

Prevalence of disease in Group 1 (*p*)	Prevalence of disease in Group 2 (*q*)	Percent increase in absolute disparity (*δp* − *δq*) > 0 (%)	Percent increase in relative disparity (*δp* − *δq*) > (*p* − *q*) *δq*/*q* (%)
0.2	0.19	52.6	95.2
0.2	0.15	62.5	80.0
0.2	0.1	75.0	66.7
0.2	0.05	87.5	57.3
0.2	0.01	97.5	51.4
0.2	0.001	99.7	50.2
0.15	0.1	66.7	74.9
0.15	0.05	83.3	60.0
0.15	0.01	96.7	51.8
0.15	0.001	99.7	50.3
0.1	0.05	75.1	66.7
0.1	0.01	95.0	52.6
0.1	0.001	99.5	50.3
0.05	0.01	90.0	55.6
0.05	0.001	99.0	50.5
0.005	0.001	90.0	55.5
0.002	0.0019	52.5	95.2
0.002	0.001	75.0	66.8

## Example

Table 2 gives the infant deaths per 1,000 live births from 1999 to 2010 for non-Hispanic Black and Non-Hispanic White populations. From 1999 to 2000, the infant mortality rate declined for both Black and White populations (*δp* < 0 and *δq* < 0). Because (*δp* − *δq*) < (*p* − *q*) *δq*/*q* < 0, both absolute and relative disparity declined from 1999 to 2000. From 2000 to 2001, the infant mortality rate for Black population declined but increased for the White population (*δp* < 0 and *δq* > 0) and both Black–White absolute and relative disparities declined. Infant mortality rate increased for both Black and White populations from 2001 to 2002 (*δp* > 0 and *δq* > 0) and since (*δp* − *δq*) > (*p* − *q*) *δq*/*q* > 0, both absolute and relative disparities increased. Infant mortality rate declined for three consecutive years during 2007–2010 for both Black and White populations. From 2007 to 2008 and from 2009 to 2010, both relative and absolute disparities for infant mortality rates declined. On the other hand, the absolute disparity declined and the relative disparity increased from 2008 to 2009 because (*p* − *q*) *δq*/*q* < (*δp* − *δq*) < 0. Overall, for the period 1999 to 2010, the mortality rates for Blacks and Whites declined by 19% and 10.2%, respectively. Both absolute and relative disparities in mortality rates between Black and White populations declined. These results show that when prevalence of disease increase or decrease simultaneously for the two populations, the increase or decrease in absolute disparity has to be sufficiently large enough to warrant a corresponding increase or decrease in relative disparity. For example, when prevalence of disease for both populations declines (*δp* < 0 and *δq* < 0), the reduction in absolute disparity has to be larger than (*p* − *q*)|*δq*|/*q* for both absolute and relative disparities to decline. Therefore, a reduction in absolute disparity still provides evidence for reducing disparities, but this reduction has to be sufficiently large enough to reduce relative disparities.

**Table 2:** Infant deaths per 1,000 live births from 1999 to 2010 for non-Hispanic Black and non-Hispanic White populations in the United States.

Year	Crude rate Black (*p*)	Crude rate White (*q*)	Change in crude rate Black (*δp*)	Change in crude rate White (*δq*)	Absolute disparity	Increase/ decrease in absolute disparity (*δp* − *δq*)	Relative disparity	Increase/ decrease in relative disparity	(*p* − *q*) *δq*/*q*
1999	14.138	5.763			8.375		1.453		
2000	13.588	5.697	−0.55	−0.066	7.891	−0.484	1.385	−0.068	−0.096
2001	13.456	5.716	−0.132	0.019	7.74	−0.151	1.354	−0.031	0.026
2002	13.886	5.799	0.43	0.083	8.087	0.347	1.395	0.041	0.112
2003	13.603	5.697	−0.283	−0.102	7.906	−0.181	1.387	−0.007	−0.142
2004	13.596	5.661	−0.007	−0.036	7.935	0.029	1.401	0.014	−0.050
2005	13.632	5.761	0.036	0.1	7.871	−0.064	1.366	−0.035	0.140
2006	13.351	5.581	−0.281	−0.18	7.77	−0.101	1.392	0.025	−0.245
2007	13.315	5.629	−0.036	0.048	7.686	−0.084	1.365	−0.027	0.067
2008	12.67	5.516	−0.645	−0.113	7.154	−0.532	1.297	−0.068	−0.154
2009	12.402	5.326	−0.268	−0.19	7.076	−0.078	1.329	0.032	−0.246
2010	11.458	5.176	−0.944	−0.15	6.282	−0.794	1.214	−0.115	−0.199

Suppose the prevalence of disease declines for the two population groups from time *t*_0_ to *t*_1_ and the disparity in prevalence between the two population groups is eliminated at time *t*_1_, then *p* + *δp* = *q* + *δq* at time *t*_1_ and the change in absolute disparity is given by (*δp* − *δq*) = − (*p* − *q*). Since 0 < |*δq*|/*q* < 1, the reduction in absolute disparity is larger than (*p* − *q*)|*δq*|/*q* and the relative disparity also declines. The change in relative disparity is given by −(*p* − *q*)/*q*. When prevalence of disease declines for both population groups, the disparity in prevalence can be eliminated only when relative disparity declines.

There is a debate about which measure (absolute or relative) to use to evaluate progress towards reducing health disparities. Measuring the absolute disparity and the size of the two population groups provide a method to estimate the population health burden of disparities between the two groups (Harper & Lynch). Since relative disparity is scale invariant, relative disparities could be used to compare disparities of health outcomes that were measured on different scales (Harper et al., 2008b). Keppel (2007) listed the ten largest health disparities for five US racial and ethnic groups by ranking the relative disparities for Healthy People 2010 objectives. However, Harper et al. pointed out that coronary heart disease and stroke mortality, which account for large reductions in life expectancy if these diseases were eliminated were not in this list (Harper et al., 2008b; Wong et al., 2002). On the other hand, it is important to use relative disparities in measuring Healthy People 2010 objectives because the overarching goal of the Healthy People 2010 initiative is to eliminate, not just reduce, health disparities. Our results imply that a reduction in absolute disparities even without a reduction in relative disparities still remains an indicator of *progress towards reduction* of disparities when prevalence of disease declines in both populations.

Other authors have pointed out that the measurement of health inequality should not only be value neutral, but should also be considered using implicit value judgements that reflects values based on the context of fairness and social acceptance (Harper et al., 2010; Asada, 2010). Kjellsson, Gerdtham & Petrie (2015) showed that for bounded variables representing health outcomes, data can be represented as attainments or shortfalls. The authors discussed two different relative measures, attainment-relative measure and shortfall-relative measure, and these two measures could have different values. They also recommended on choosing between an attainment-relative, absolute, or shortfall-relative measure based on a value judgement.

## Conclusion

The trends for absolute disparity and relative disparity between two population groups could move in opposite directions when the prevalence of disease in each population either increases or decreases simultaneously. When using these two disparity measures to analyze trends in disparity, the trends can be expected to move in opposite directions when the prevalence of disease in the advantaged group is very much lower relative to the prevalence of disease in the disadvantaged group. When prevalence increases in both population groups, there are *three* possible scenarios:

(1) Both absolute disparity and relative disparity increase.
(2) Absolute disparity increases but relative disparity declines. The increase in absolute disparity is not large enough to also increase relative disparity.
(3) Absolute disparity declines. This implies that relative disparity also declines.

When prevalence declines in both population groups, the three possible scenarios are given below:

(1) Absolute disparity increases. This implies that relative disparity also increases and this is a situation we have to avoid when the overall population health improves.
(2) Absolute disparity declines but relative disparity increases. This is a situation that can be considered to reflect some progress towards reducing disparities, but the reduction in absolute disparity is not large enough to also reduce relative disparity.
(3) Both absolute and relative disparities decline. This is the best scenario in that it not only shows progress towards reduction in disparities but, assuming the pattern will continue into the future, it also shows progress towards the elimination of disparities.

When evaluating interventions to reduce health disparities, it is important to consider both the absolute and relative disparity measures and all the scenarios discussed in this paper. Absolute measures may be most useful when the purpose of the evaluation is to assess progress towards reduction of disparity i.e., the magnitude of the disparity is decreasing. Whereas, the relative measure may have greater utility when the purpose of the evaluation is to determine whether a disparity is being eliminated.

## Additional Information and Declarations

### Data Availability

The following information was supplied regarding data availability: The research in this article did not generate any raw data.

## References

[ref-1] Agency for Health Care Research and Quality (2013). National healthcare disparities report. http://www.ahrq.gov/research/findings/nhqrdr/nhdr13/2013nhdr.pdf.

[ref-2] Andrulis DP, Siddiqui NJ, Purtle JP, Duchon L Patient protection and affordable care act of 2010: advancing health equity for racially and ethnically diverse populations. Joint Center for Political and Economic Studies.

[ref-3] Asada Y (2010). On the choice of absolute or relative inequality measures. Milbank Quarterly.

[ref-4] Bhopal RS (2008). Re: “an overview of methods for monitoring social disparities in cancer with an example using trends in lung cancer incidence by area-socioeconomic position and race-ethnicity, 1992–2004”. American Journal of Epidemiology.

[ref-5] Centers for Disease Control and Prevention (2013). CDC Health disparities and inequalities report—United States, 2013. MMWR Supplement Vol. 62/ No. 3. http://www.cdc.gov/mmwr/pdf/other/su6203.pdf.

[ref-6] Harper S, King NB, Meersman SC, Reichman ME, Breen N, Lynch J (2010). Implicit value judgements in the measurement of health inequalities. Milbank Quarterly.

[ref-7] Harper S, Lynch J University of Michigan. Methods for measuring cancer disparities: using data relevant to Healthy People 2010 cancer-related objectives. http://seer.cancer.gov/archive/publications/disparities/measuring_disparities.pdf.

[ref-8] Harper S, Lynch J, Meersman SC, Breen N, Davis WW, Reichman ME (2008a). An overview of methods for monitoring social disparities in cancer with an example using trends in lung cancer incidence by area-socioeconomic position and race-ethnicity, 1992–2004. American Journal of Epidemiology..

[ref-9] Harper S, Lynch J, Meersman SC, Breen N, Davis WW, Reichman ME (2008b). Harper et al. respond to “Measuring social Disparities in Health”. American Journal of Epidemiology.

[ref-10] Houweling TA, Kunst AE, Huisman M, Mackenbach JP (2007). Using relative and absolute measures for monitoring health inequalities: experiences from cross-national analyses on maternal and child health. International Journal for Equity in Health.

[ref-11] Institute of Medicine (2002). Unequal treatment: confronting racial and ethnic disparities in healthcare.

[ref-12] Keppel KG (2007). Ten largest racial and ethnic health disparities in the United States based on Healthy People 2010 objectives. American Journal of Epidemiology.

[ref-13] Keppel KG, Pearcy JN, Heron MP (2010). Is there progress toward eliminating racial/ethnic disparities in the leading causes of death?. Public Health Reports.

[ref-14] Kjellsson G, Gerdtham UG, Petrie D (2015). Lies, damned lies, and health inequality measurements: understanding the value judgements. Epidemiology.

[ref-15] LaVeist TA, Gaskin D, Richard P (2011). Estimating the economic burden of racial health inequalities in the United States. International Journal of Health Services.

[ref-16] Lorenc T, Petticrew M, Welch V, Tugwell P (2013). What types of interventions generate inequalities? Evidence from systematic reviews. Journal of Epidemiology and Community Health.

[ref-17] Lynch J, Harper S (2005). University of Michigan, Part II—issues in measuring health disparities. https://open.umich.edu/sites/default/files/1232/lectures-1/Part-II.pdf.

[ref-18] Messer LC (2008). Invited commentary: measuring social disparities in health—what was the question again?. American Journal of Epidemiology.

[ref-19] US Department of Health and Human Services (HHS) (2011). HHS action plan to reduce racial and ethnic health disparities. http://minorityhealth.hhs.gov/npa/files/Plans/HHS/HHS_Plan_complete.pdf.

[ref-20] US Department of Health and Human Services (2015). Healthy People 2020.

[ref-21] Wong MD, Shapiro MF, Boscardin WJ, Ettner SL (2002). Contribution of major diseases to disparities in mortality. New England Journal of Medicine.

